# FOUR LIPS and MYB88 conditionally restrict the G1/S transition during stomatal formation

**DOI:** 10.1093/jxb/ert313

**Published:** 2013-10-11

**Authors:** EunKyoung Lee, Xuguang Liu, Yana Eglit, Fred Sack

**Affiliations:** ^1^Department of Botany, University of British Columbia, Vancouver V6T 1Z4, Canada

**Keywords:** Endoreplication, FOUR LIPS, microtubules, mitosis, S-phase.

## Abstract

Consistent with their valve-like function in shoot–atmosphere gas exchange, guard cells are smaller than other epidermal cells and usually harbour 2C DNA levels in diploid plants. The paralogous *Arabidopsis* R2R3 MYB transcription factors, FOUR LIPS and MYB88, ensure that stomata contain just two guard cells by restricting mitosis. The loss of both FLP and MYB88 function in *flp myb88* double mutants induces repeated mitotic divisions that lead to the formation of clusters of stomata in direct contact. By contrast, CYCLIN DEPENDENT KINASE B1 function is required for the symmetric division that precedes stomatal maturation. It was found that blocking mitosis by chemically disrupting microtubules or by the combined loss of FLP/MYB88 and CDKB1 function, causes single (undivided) guard cells (sGCs) to enlarge and attain mean DNA levels of up to 10C. The loss of both FLP and CDKB1 function also dramatically increased plastid number, led to the formation of multiple nuclei in GCs, altered GC and stomatal shape, and disrupted the fate of lineage-specific stem cells. Thus, in addition to respectively restricting and promoting symmetric divisions, FLP and CDKB1 together also conditionally restrict the G1/S transition and chloroplast and nuclear number, and normally maintain fate and developmental progression throughout the stomatal cell lineage.

## Introduction

Cell differentiation is often associated with defined DNA levels that contribute to the function of specialized cell types (Nagl, 1976; Lee *et al.*, 2009). While many plant cells contain 2C DNA levels throughout their lifespan, others undergo varying degrees of endoreplication meaning DNA replication without mitosis (Lee *et al.*, 2009). Examples of cells and tissues that endoreplicate during development include trichomes, endosperm, epidermal pavement cells in leaves and in the hypocotyl, and suspension cultures (Sugimoto-Shirasu and Roberts, 2003; Lee *et al.*, 2009).

Endoreplicaton occurs either through endocycling or endomitosis. In endocycling, G and S phases occur but not mitotic entry. The latter does take place in endomitosis, and chromosomes start to condense. However, the chromosomes fail to separate and, instead, the cells revert to a phase resembling G1 (Lee *et al.*, 2009). In addition, endomitosis can lead to nuclear division without cytokinesis, resulting in the formation of several nuclei in one cell (Meserve and Duronio, 2012; Pandit *et al.*, 2012).

DNA levels in endoreplicated cells correlate strongly with cell size (Melaragno *et al.*, 1993; Kondorosi *et al.*, 2000; Sugimoto-Shirasu and Roberts, 2003). Moreover, endoreplication can be essential for cell fate acquisition and maintenance as well as for patterning and distributing specialized types of cells (Bramsiepe *et al.*, 2010; Roeder *et al.*, 2010).

Stomata consist of two guard cells (GCs) around a pore, a configuration central to the regulation of shoot–atmosphere gas exchange. *Arabidopsis* stomata develop from asymmetric divisions of lineage-specific stem cells with the smaller daughter cell, the meristemoid, later developing into a Guard Mother Cell (GMC) precursor (Bergmann and Sack, 2007). The GMC then divides just once which ensures that mature stomata each consist of just two GCs. Diploid *Arabidopsis* accessions contain GCs that also harbour 2C DNA levels, but adjacent pavement cells in leaves often endoreplicate and reach 16C to 32C DNA levels (Galbraith *et al.*, 1991; Melaragno *et al.*, 1993; see Supplementary Fig. S1E at *JXB* online).

During *Arabidopsis* leaf development, a complex containing CDKB1;1 and CYCLINA2;3 (CYCA2;3) promotes division and restricts endoreplication in many cells (Boudolf *et al.*, 2004*b*, 2009; Imai *et al.*, 2006). However, excessive endoreduplication takes place in whole organs when cyclin-dependent kinase (CDK) activity is inhibited (De Veylder *et al.*, 2011).

During stomatal development, several *CDKB1* and *CYCA2* genes promote the symmetric division of the GMC precursor, and thus are critical for constructing the mature stomatal valve. The loss-of-function of both *CDKB1;1* and *CDKB1;2* in double mutants or in a dominant negative form of *CDKB1* (*pro35S:CDKB1;1 N161*) blocks GMC division and induces the formation of abnormal single guard cells (Boudolf *et al.*, 2004*a*; Xie *et al.*, 2010). Single guard cells (sGCs) also arise when the function of three *CYCA2* genes is compromised (*cyca2;2*, *cyca2:3*, and *cyca2;4*) (Vanneste *et al.*, 2011; see Supplementary Fig. S1A–D at *JXB* online).

The transcription of the *CDKB1;1* and *CYCA2;3* genes during stomatal development is regulated in part by the *FOUR LIPS* (*FLP*) gene which encodes an R2R3 MYB protein (Xie *et al.*, 2010; Vanneste *et al.*, 2011). Loss-of-function mutations in *FLP* induce ectopic and extra symmetric divisions that produce clusters of GCs and stomata in direct contact (see Supplementary Fig. S1A–D at *JXB* online). The *MYB88* gene, which is an *FLP* paralogue, shows no loss-of-function phenotype on its own, but acts synergistically in a *flp myb88* double mutant by increasing symmetric divisions and stomatal cluster size (Lai *et al.*, 2005). In a *flp myb88 cdkb1;1 cdkb1;2* quadruple mutant, the *cdkb1;1 cdkb1;2* phenotype is epistatic to that of *flp-1 myb88* resulting in many sGCs that are oval-shaped in face view and that lack a dividing wall (Xie *et al.*, 2010).

It is shown here that guard cells exhibit high levels of endoreplication when the loss of *FLP* and *MYB88* function in restraining Guard Mother Cell division is combined with blocked mitosis. Therefore, these MYB proteins can limit S-phase entry as well as mitosis. Moreover, the loss of these combined functions leads to the fate disruption of several types of epidermal cells and induces the abnormal expression of a stomatal lineage stem cell gene.

## Materials and methods

### Plant materials

All the *Arabidopsis* lines used were in the Columbia (Col-0) ecotype including the *flp-1 myb88* and *cdkb1;1 cdkb1;2* double mutants, and the *flp-1 myb88 cdkb1;1 cdkb1;2* quadruple mutant (Lai *et al.*, 2005; Xie *et al.*, 2010). Plants were grown on sterile agar or soil (Promix, Premier Brands) at 22 °ºC with a 16/8h light/dark cycle.

### Reporter constructs

The *proFAMA:GFP* transcriptional fusions were generated by PCR amplification of 3423bp of upstream sequence of the start codon (see Supplementary Table S1 at *JXB* online), followed by cloning the PCR products into the *pENTR/TOPO* vector (Invitrogen, Carlsbad, CA), and then by recombination into the *pGWB4* destination binary vector (Tanaka *et al.*, 2011). The *proFAMA:GFP* construct was transformed into wild-type plants (*Agrobacterium* strain GV3101; Clough and Bent, 1998). Transgenic lines were selected on half-strength MS medium containing 25 µg ml^–1^ hygromycin.

### Measurement of epidermal cell size

To reduce growth variations, different lines were sown at the same time on plates containing half- strength MS medium for each experiment. Cotyledons were harvested 21 d after germination. For clearing, after a water rinse, cotyledons were fixed in acidified methanol (containing 20% methanol and 4% concentrated hydrochloric acid) for 15min in a hot (57 °C) water bath. The acidified methanol was then replaced with a basic solution (7% m/v sodium hydroxide in 60% ethanol) for 30min at room temperature. Samples were then rehydrated in a series of ethanol solutions 40%, 20%, 10%, and incubated for at least 30min at each step. Tissues were then placed in a mixture of 5% ethanol and 25% glycerol for storage at room temperature. The abaxial cotyledon epidermis was visualized using an Olympus AX-70 wide-field light microscope. For sampling, images were captured at two positions along the length of the cotyledon, at one-quarter and at three-quarters of the distance from the tip to the base of the cotyledon. Six images were collected from each cotyledon and six cotyledons were counted for each genotype. Cell areas were measured using ImageJ software.

### Phenotypic quantification

The total number of stomatal ‘units’ (SUs) were scored. The ‘units’ can include a normal stoma as well as a group of stomata in direct contact. SU number was scored from differential interference contrast light micrographs of the abaxial epidermis of entire cotyledons. The genotypes analysed included the Col-0 wild type, a *flp-1 myb88* double mutant, a *cdkb1;1 cdkb1;2* double mutant, and a *flp-1 myb88 cdkb1;1 cdkb1;2* quadruple mutant. Values are means ±SE.

### Measurement of nuclear DNA content

Cotyledons were harvested from seedlings grown on plates containing half-strength MS medium and fixed in a solution of 9:1 (v/v) ethanol:acetic acid at –20 °C for at least 1h. For staining, tissues were rinsed once in TN buffer (Tris–NaCl buffer, 50mM sodium chloride, 100mM Tris–hydrochloric acid, pH 8.0) and then stained in the dark for 24h in TN buffer containing 0.1 µg ml^–1^ 4′6-diamidino-2-phenylindole (DAPI).

Nuclear DAPI fluorescence was visualized using mercury lamp excitation. The relative nuclear DNA content was calculated by the total gray values of the nuclear zone using ImageJ software (Boudolf *et al.*, 2004*a*). Statistical differences for relative DNA content were determined using a non-parametric test (Mann Whitney test) and Graphpad Prism 5.0 software (San Diego, California, USA).

Cell shape and position were evaluated by bright-field microscopy. To standardize image capture, all fluorescence images from different samples were obtained using comparable microscope settings.

### Flow cytometry

First leaves from plants grown for 21 d after germination were harvested from seedlings grown on half-strength MS medium plates. Seeds were sown simultaneously. Nuclei were isolated according to Suda and Travnicek (2006) with only slight modifications. About 20mg of cotyledon tissue was minced with a razor in 600 µl OttoI solution (0.1M citric acid monohydrate, 0.5% v/v TWEEN 20, 0.1% v/v β-mercaptoethanol). The solution was filtered with Miracloth and a 22 µm micropore membrane to separate nuclei from cell and tissue debris. The supernatant was centrifuged at 3000rpm for 3min to spin down nuclei and the pellet was re-suspended in 150 µl OttoI solution. Then 600 µl of OttoII solution (0.4M sodium phosphate dibasic dodecahydrate, 0.05mg ml^–1^ propidium iodide, 1mg ml^–1^ RNase) was added to the re-suspended nuclei. Suspensions were analysed using a FACScan flow cytometer (Becton Dickinson). The experiments were repeated independently two or three times with comparable results. The output graph was processed using flowJo software.

### RNA isolation and quantitative real-time PCR

Total RNA was extracted from 15–18-d-old Col-0, *flp-1 myb88, cdkb1;1 cdkb1;2*, and *flp-1 myb88 cdkb1;1 cdkb1;2* cotyledons to quantify the expression of cell cycle-related genes using a Plant RNeasy Kit (Qiagen). cDNA was synthesized from 1 µg of DNase-treated total RNA using an oligo (dT)_18_ primer and superscript III reverse transcriptase enzyme (Invitrogen, Carlsbad, CA, USA). One microlitre of RT product was used for quantitative real-time PCR that used an iQ SYBR Green Supermix (Bio-Rad, Hercules, CA), and then was analysed using an iQ5 real-time PCR machine (Bio-Rad). The *Arabidopsis ACTIN2* gene was used to approximate relative levels of constitutive and normalized control expression. Relative changes in gene expression were quantified using the 2^–ΔΔCT^ method (Livak and Schmittgen, 2001) with data obtained from at least two biological replicates. Gene-specific primers are shown in Supplementary Table S1 at *JXB* online.

### Oryzalin treatment

Oryzalin (Sigma) was dissolved in dimethylsulphoxide (DMSO) at 25mM (stock solution), stored at –20 °C, and diluted with sterilized water to 25 µM. Cotyledons from 3-d-old seedlings were submerged in this oryzalin solution for 24h and then removed 1 d later. Plants were then rinsed three times with sterilized water and then cultivated in a growth chamber for 3–5 d. DMSO was used as a control. These experiments were repeated independently three times with similar results.

### Microscopy

GFP and propidium iodide fluorescence were viewed and captured using a Nikon Eclipse 80i confocal scanning laser microscope and laser lines 488nm and 543nm with Nikon Plan Apochromat oil immersion objectives (40×1.0 NA, and 60×1.4 NA). An Olympus AX-70 wide-field light microscope was used to capture Differential Interference Contrast images. To visualize nuclear DAPI fluorescence, two-photon microscopy was performed using a Mai-Tai titanium sapphire laser (Spectra-Physics Lasers) on a Zeiss Meta 510 Laser Scanning Confocal Microscope using an excitation spectrum of 760–900nm.

## Results

### Chemical disruption of microtubules induces GC endoreplication in a myb double mutant

To probe the role of microtubule (MT) function in stomatal development, MT depolymerizing drugs were applied to developing *Arabidopsis* cotyledons and leaves. Oryzalin is a dinitroaniline herbicide that depolymerizes microtubules, a disruption that can arrest division in the M-phase (Morejohn *et al.*, 1987).

**Fig. 1.**
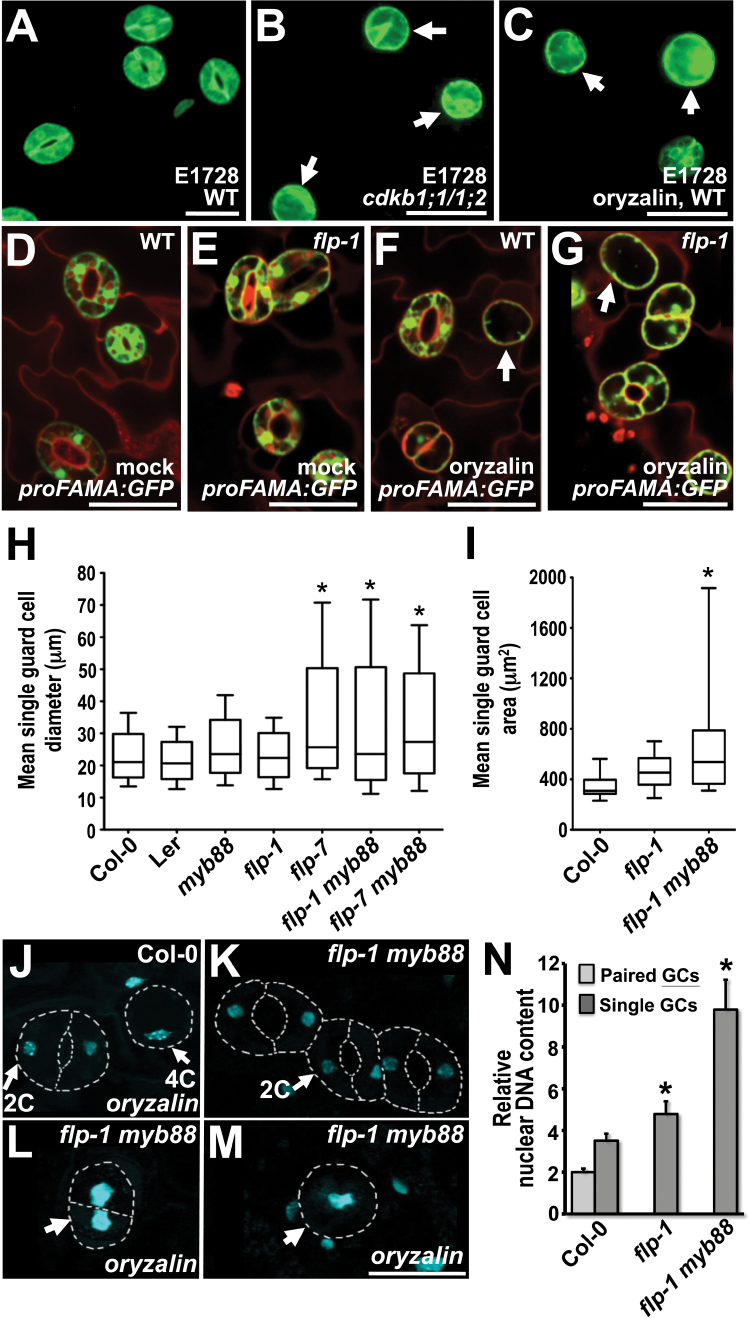
Chemical disruption of mitosis induces enlarged and highly endoreplicated single guard cells (sGCs) in *flp myb88* mutants. (A) Wild-type stomata expressing the mature guard cell fate marker, E1728. (B) *cdkb1;1 cdkb1;2* sGCs expressing E1728 (arrows). (C) Oryzalin-induced sGCs in wild-type also express E1728. (D–G) *proFAMA:GFP* expression which also marks GC fate. (D) Wild-type control (DMSO treatment). (E) *flp-1* mutant background (DMSO). (F) Wild-type stomata treated with oryzalin for 24h showing an undivided sGC (arrow). (G) *flp-1* treated with oryzalin showing a sGC (arrow). (H) Mean GC diameter 6 d after treatment with 25 µM oryzalin for 24h as a function of genotype. The diameters of normal GCs as well as abnormal sGCs were scored. Genotypes ranked from lowest to highest diameters (bars). (I) Mean GC area after oryzalin treatment. Three-day-old Col-0, *flp-1*, and *flp1 myb88* seedlings treated and scored as in (H). Genotypes ranked from lowest to highest values (bars). Data in (H) and (I) presented as box plots, in which the box encompasses data for the 25th to 75th percentiles. The horizontal line within each box is the median (50%). Error bars represent the 5th (lower bar) and the 95th (upper bar) percentiles. (A–G) Scale bars=25 µm. Stars (*) denote means significantly different from the wild type (*P* <0.005). (J–N) Cotyledons from Col-0, *flp-1*, and *flp-1 myb88* plants were treated with oryzalin (or DMSO control) for 24h after staining with 4′6-diamidino-2-phenylindole (DAPI). Nuclear fluorescence was imaged using wide-field microscopy. Whereas sGCs that form after oryzalin treatment display mostly 4C levels in wild-type plants, in *flp1 myb88* this mean was 10C. (J–M) Micrographs showing examples of normal and enlarged guard cell nuclei. Stomatal outlines indicated by dashed lines. Arrows indicate nuclei in guard cells that are enlarged compared with other nuclei shown and that probably contain 4C DNA levels. (J–M) Bars=25 µm. (J) Stomata from wild-type (Col-0) plants treated with oryzalin. Left: probably 2C guard cells in a normal stoma. Right: a sGC with a larger nucleus likely to be 4C. (K) Stomatal cluster in a *flp-1 myb88* cotyledon that was not treated with oryzalin containing 2C DNA levels in guard cells. (L, M), *flp-1 myb88* cotyledons treated with oryzalin. Arrows indicate enlarged nuclei. (L) Nuclei are enlarged in both GC-like cells. (M) Enlarged nucleus in sGC. Stomatal morphogenesis appears to be blocked or delayed. (N) Graph showing nuclear sizes in Col-0, *flp-1*, and *flp-1 myb88* plants treated with oryzalin. Relative DNA levels quantified by the areas of nuclei/gray values derived from DAPI-fluorescence. Only single guard cells were sampled in *flp-1* and *flp-1 myb88*. Sample sizes: Col-0: 145 paired GCs and 65 sGCs; *flp-1*: 59 sGCs; 48 *flp-1 myb88* sGCs. Bars, means ±SE. Stars (*) denote means significantly different from the wild type (*P* <0.001). (This figure is available in colour at *JXB* online.)

While many stomata appear unaffected by oryzalin treatment, a fraction formed undivided, round-to-oval-shaped single guard cells (sGCs). Thus, GMC precursor cells exposed to oryzalin eventually acquire a mature stomatal fate, as shown by the expression of the guard cell fate markers E1728 and *proFAMA:GFP* (Fig. 1C, D, F). This sGC phenotype closely resembles that of the *cdkb1;1 cdkb1;2* double mutants (Fig. 1B).

Since *FLP* and *MYB88* restrict GMCs to a single mitotic cell cycle, the effects of oryzalin application were analysed in various *flp* and *myb88* mutant backgrounds. Treatment with oryzalin of *flp-1* and *flp-7* (a stronger allele) single mutants and of *flp-1 myb88* and *flp-7 myb88* double mutant plants suppressed the excess division (cluster) phenotype and instead induced the formation of sGCs. Unlike the stomatal clusters found in mutant plants, these sGCs are correctly patterned.

Single GCs in various combinations of *flp/myb88* alleles and mutants were about 50% larger compared with those in oryzalin-treated wild-type plants with respect to sGC area and diameter (Fig. 1F–I). Similarly, the largest sGCs (maximal diameter) were twice as large (72 versus 36 µm) in *flp-1 myb88* compared with the wild type (Fig. 1H).

To analyse the relationship between GC size and nuclear DNA content, gray-scale values derived from DAPI (4′,6-diamidino-2-phenylindole) fluorescence were measured using wide-field microscopy. Although pavement cells in the *Arabidopsis* epidermis undergo varying degrees of endoreplication, stomata in the Col-0 ecotype harbour 2C levels of DNA (Melaragno *et al.*, 1993). As expected, many single GCs induced by oryzalin in wild-type plants displayed roughly double the DNA content of normal GCs (Fig. 1J–N). This doubling is consistent with oryzalin blocking the G2-to-M transition but not S-phase progression. These 4C DNA levels after oryzalin treatment are comparable with those in sGCs induced by transformation with a *CDKB1;1 N161* dominant negative construct (Boudolf *et al.*, 2004*a*) (Fig. 1N).

In contrast to treatment of *N161* plants, oryzalin dramatically increased nuclear DNA levels in sGCs in a various *flp myb88* mutant combinations. Whereas the Col-0 wild-type exhibited mostly 4C levels, sGCs in a *flp1 myb88* double mutant harboured a mean DNA content of 10C when treated with oryzalin (Fig. 1N). By contrast, oryzalin did not affect sGC size in *cdkb1;1 cdkb1;2* double mutants (see Supplementary Fig. S2 at *JXB* online). Thus, the substantive enlargement of drug-induced sGCs depends primarily upon the combined loss of *FLP/MYB88* function.

### Combined loss of FLP/CDKB1 function induces guard cell and nuclear enlargement

**Fig. 2.**
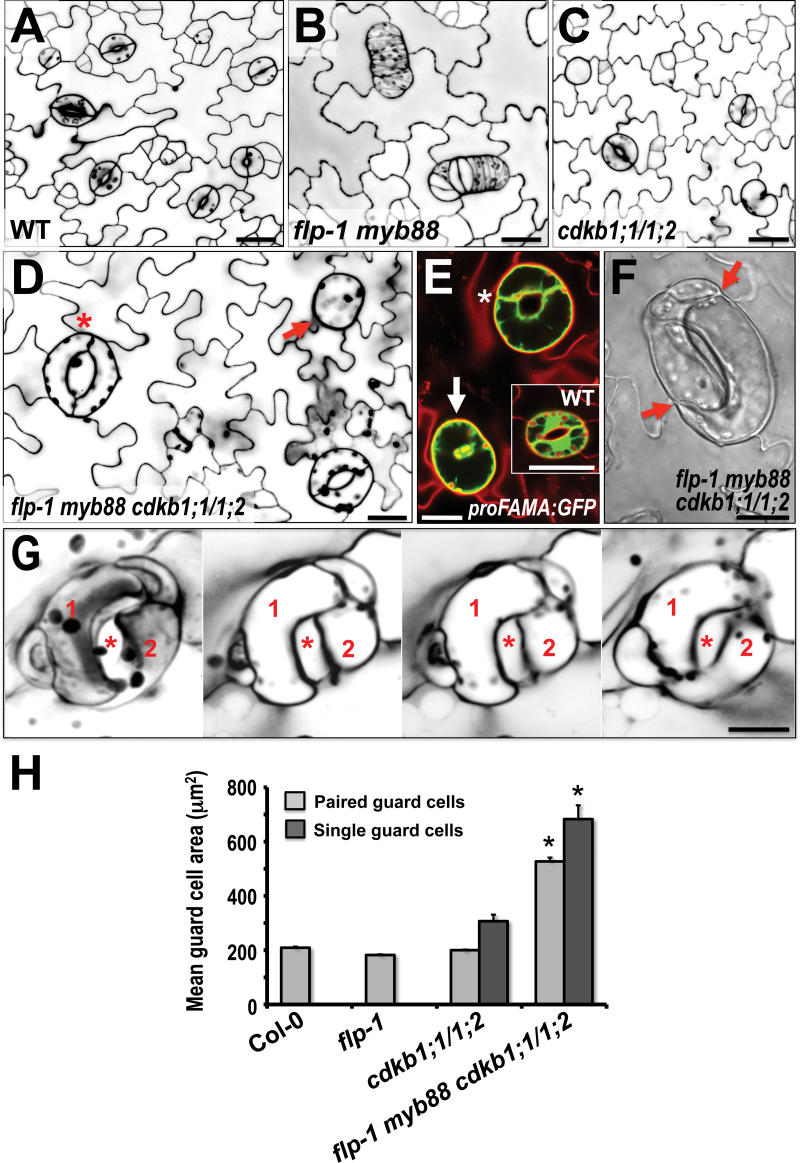
Combined loss of *FLP/MYB88* and *CDKB1;1/1;2* function increases stomatal size and disrupts GC and stomatal shape. (A) Wild-type (WT) stomata. (B) *flp-1 myb88* stomatal clusters. (C) *cdkb1;1 cdkb1;2* stomata. Single, oval-shaped GC shown at upper left. Lower right: kidney-shaped single GC. (D) *flp-1 myb88 cdkb1;1 cdkb1;2* background showing enlarged GCs in an otherwise normal stoma (*) as well as an enlarged sGC upper right (red arrow). (E) *proFAMA:GFP* expression indicates stomatal identity in sGC (arrow, lower left) as well as in an abnormally shaped stoma (*) in *flp-1 myb88 cdkb1;1 cdkb1;2*. First leaf from a 15-d-old plant. Inset: normal stoma in a wild-type plant. (F) Abnormal guard cell enlargement, twisting (red arrows), and loss of kidney shape. From a 20-d-old *flp-1 myb88 cdkb1;1 cdkb1;2* quadruple mutant. (G) Four successive optical sections of the same abnormal stoma. Guard cells 1 and 2 are labelled in red. Star marks an abnormal and twisted stomatal pore. From a 20-d-old *flp-1 myb88 cdkb1;1 cdkb1;2* cotyledon. (H) Quantification of guard cell areas measured from six, 21-d-old cotyledons. Sample sizes: Col-0 (225), *flp-1* (225), *cdkb1;1/1;2* (225 paired GCs and 164 sGCs), and *flp-1 myb88 cdkb1;1 cdkb1;2* (225 paired GCs and 182 sGCs). Bars, means ±SE. Stars indicate values significantly different from the wild type (*P* <0.001). All figures in (A) to (E) are at same magnification and from 12-d-old first leaves. Cell walls in all figures (except F) were visualized with propidium iodide fluorescence. All scale bars=20 µm. (This figure is available in colour at *JXB* online.)

To explore the effects of the loss of both MYB and CDKB1 function on stomatal size and morphology, a *flp-1 myb88 cdkb1;1 cdkb1;2* quadruple mutant was used. In addition to exhibiting many relative large sGCs, this combined loss-of-function background also induced some stomata to enlarge considerably in mature leaves and cotyledons (Fig. 2). By contrast, guard cells in wild-type, *flp-1*, and in *cdkb1;1 cdkb1;2* double mutant plants were much smaller than in the quadruple mutant (Fig. 2H).

**Fig. 3.**
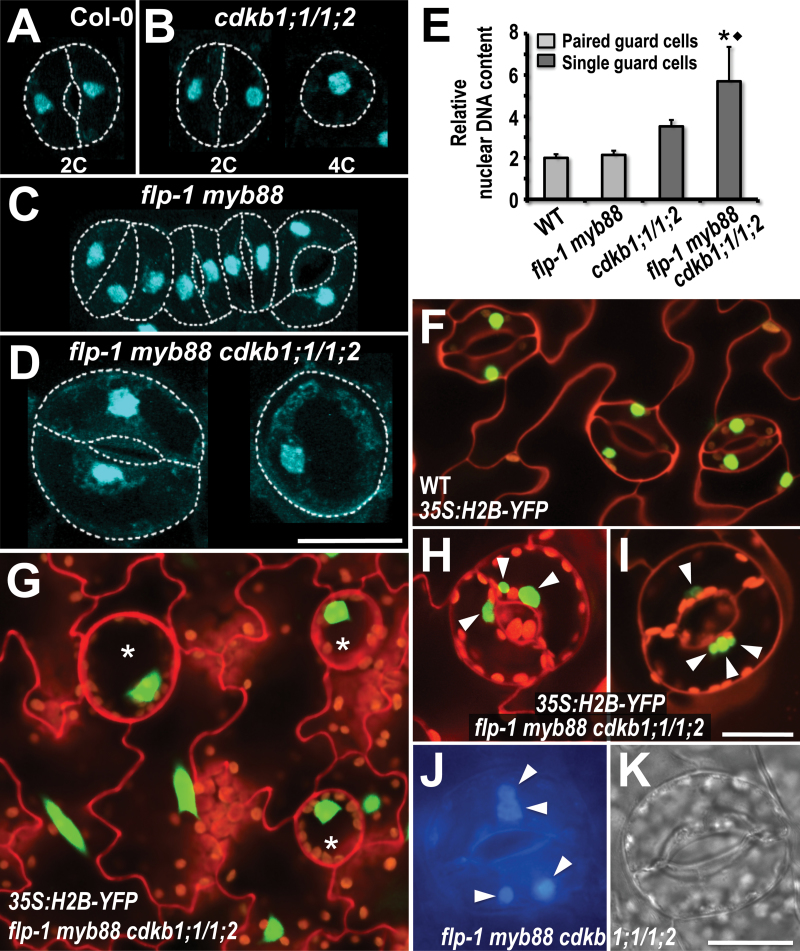
Guard Cell ploidy increases substantively in a *flp-1 myb88 cdkb1;1 cdkb1;2* quadruple mutant that also harbours some cells with multiple nuclei. Quantification of guard cell relative nuclear DNA content in cotyledons of Col-0, *flp-1*, *flp-1 myb88*, and *flp-1 myb88 cdkb1;1 cdkb1;2* plants visualized with DAPI. White dashes indicate stomatal outline. (A) Wild-type stoma. (B) Normal stoma (left) and a single guard cell (right) in a *cdkb1;1 cdkb1;2* double mutant background. (C) *flp-1 myb88* stomatal cluster. Relative nuclear DNA content appears roughly similar in all cells. (D) *flp-1 myb88 cdkb1;1 cdkb1;2* quadruple mutant shows enlarged nuclei in a normally-shaped stoma (left) as well as in a single guard cell (right). (E) Quantification of relative nuclear DNA content in Col-0, *flp-1*, *flp-1 myb88*, and in *flp-1 myb88 cdkb1;1 cdkb1;2* plants as measured by total gray values of DAPI-stained nuclei in guard cells of Col-0 (*n*=216), *flp-1 myb88* (*n*=216), *cdkb1;1 cdkb1;2* (*n*=51 single guard cells), and *flp-1 myb88 cdkb1;1 cdkb1;2* (*n*=68 single guard cells). Bars: means ±SE. The symbols * and ^♦^ denote values significantly different from the wild type at *P* <0.001, and from the *cdkb1;1 cdkb1;2* double mutant at *P* <0.005, respectively. Single GCs in the quadruple mutant display roughly 6C levels (mean). (F–I) Nuclear fluorescence from *35S:Histone2B-YFP*. (F) Wild-type. (G) Nuclei in single GCs (stars) in the *flp-1 myb88 cdkb1;1 cdkb1;2* quadruple mutant are much larger than in those of the wild-type. (H, I) Some GCs display multiple nuclei of different sizes (arrowheads) in quadruple mutant. (J, K) multiple nuclei (arrowheads) in GCs of quadruple mutant stained with DAPI (J). (K) is a DIC image of cells shown in (J). All figures within the same group are at the same magnification, i.e. in (A–D), (F–I), and (J, K), respectively. (A) Bar =25 µm; (J, K)=20 µm. (This figure is available in colour at *JXB* online.)

These size differences extend to nuclei. Single GCs in a *flp-1 myb88 cdkb1;l cdkb1;2* quadruple mutant harbour abnormally large nuclei with a mean DNA level of 6C (Fig. 3A–E). Because high GC ploidies were only detected in the *flp-1 myb88 cdkb1;1 cdkb1;2* quadruple mutant, endoreduplicaion is only likely to ensue when both sets of genes (*FLP/MYB88* and *CDKB1;1/CDKB1;2*) are non-functional. These data are consistent with genetically blocked mitosis (loss of *CDKB1* function) inducing cell-type specific endoreplication when excess mitotic divisions (loss of MYB function) are also precluded.

This increase in nuclear size in large GCs of the quadruple mutant was also visualized using histone H2B fused to fluorescent GFP, which marks nuclei (Fig. 3F–I). Surprisingly, fluorescence from this construct revealed the occasional presence of multiple (2–3) nuclei in large but normally shaped guard cells (Fig. 3H–K). This finding is consistent with the presence of endomitosis, as well as endocycling, during GC enlargement in *flp-1 myb88 cdkb1;1 cdkb1;2*.

### Reduced stomatal production in the quadruple mutant

**Fig. 4.**
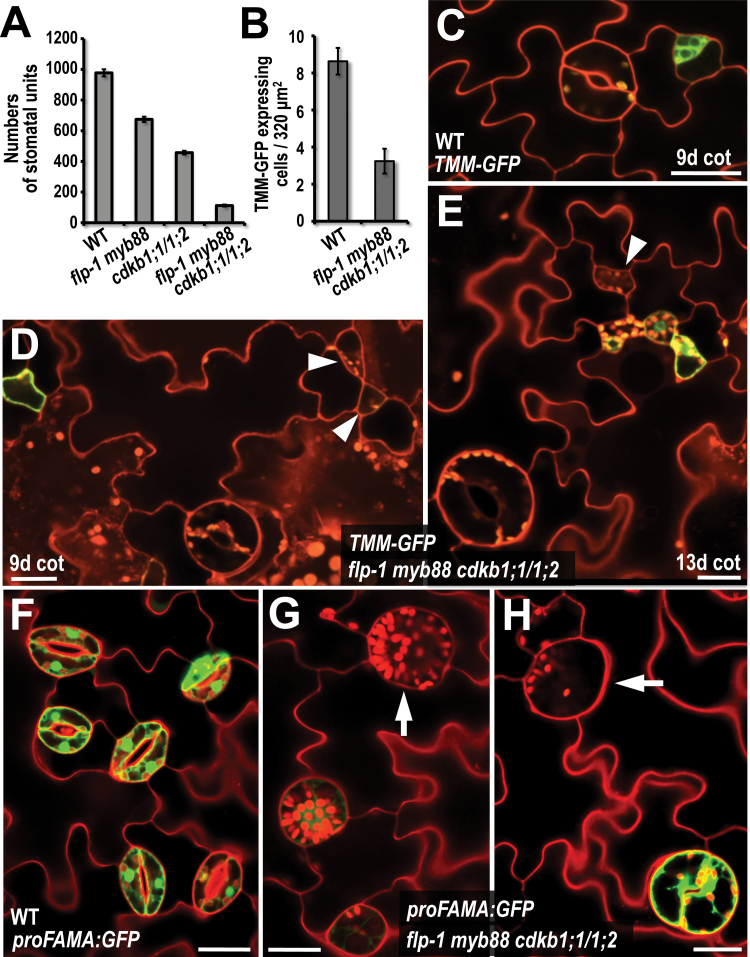
Cell fate is disrupted in the *flp-1 myb88 cdkb1;1 cdkb1;2* quadruple mutant. (A) Numbers of stomata or sGCs (both referred to as ‘stomatal units’) in wild type, quadruple mutant, *flp-1 myb88*, and *cdkb1;1 cdkb1;2*. Bars, mean ±SE. Scored from six independent samples per genotype, 15-d-old cotyledons. (B) Number of cells expressing *proTMM:TMM-GFP* (*TMM-GFP*) scored from a 320 µm^2^ region of the wild type (*n*=97) and the *flp-1 myb88 cdkb1;1 cdkb1;2* quadruple mutant (*n*=234). Bars, means ±SE. Scored from 9-d-old cotyledons, six different samples per genotype. (C) *TMM-GFP* in the wild type showing normal expression in an asymmetric division (upper right). (D, E) Cells that are comparable in size to a meristemoid and that appear to have formed in an asymmetric division vary in whether they express *TMM-GFP*. Arrowheads, meristemoid-like cells that lack GFP signal. Other small cells in the same fields display fluorescence. However, few meristemoid-like cells are normal in shape. *TMM-GFP* in 9 and 13-d-old cotyledons in the quadruple mutant. Note the abnormal shape and enlargement of stoma at the lower left in (E). (F) Wild type showing *proFAMA:GFP* fluorescence in mature stomata from a 14-d-old cotyledon. (G, H) As in (F), except in a quadruple mutant. Arrows indicate large cells that likely developed from single guard cells but that mostly (except, for example, H lower right) do not express *proFAMA:GFP.* All scale bars=20 µm. (This figure is available in colour at *JXB* online.)

The total number of stomata or stomatal ‘units’, which here refers to normal stomata as well as sGCs, was significantly lower in the *flp-1 myb88 cdkb1;1 cdkb1;2* quadruple mutant than in the wild type (Fig. 4A). Stomatal number in *Arabidopsis* correlates closely with the extent of expression of the *TOO MANY MOUTHS* (*TMM*) gene. *TMM* encodes a receptor-like protein, and its expression specifically marks the stomatal stem cell compartment which includes meristemoids and their sister cells produced by divisions that are asymmetric in size and cell fate (Fig. 4B; Nadeau and Sack, 2002). Consistent with a decreased number of mature stomata formed in the quadruple mutant, the number of stem cells, as marked by *TMM* expression, was considerably reduced (Fig. 4A, B; see Supplementary Fig. S3 at *JXB* online). Thus the combined loss of *FLP/MYB88* and *CDKB1;1/CDKB1;2* function inhibits the number of stomatal lineage-specific stem cells that form.

### The loss of MYB/CDKB1 function also disrupts GC shape and fate

Strikingly, guard cell shape and size were severely disrupted in this quadruple mutant (Fig. 2E–G). Many normally shaped guard cells later lost their shape and grew over the original edges of the GCs. No such shape changes were found in *flp my88* or in *ckdb1;1 ckdb1;2* double mutants (Fig. 2A–C), or following oryzalin treatment (Fig. 1). These abnormally shaped and enlarged cells still maintained a guard cell fate as shown by their expression of stomatal fate markers (Fig. 2E). However, this distortion of guard cell morphology probably impairs stomatal function since overall bilateral symmetry is compromised, and pore apertures and gaseous diffusion pathways become contorted, restricted or blocked. Consistent with these defects, shoot growth slowed and seed production was reduced during ageing (see Supplementary Fig. S4 at *JXB* online). Root growth was also severely reduced in the quadruple mutant (see Supplementary Fig. S4 at *JXB* online).

Cell fate and gene expression were also at least partially altered in the *flp-1 myb88 cdkb1;1 cdkb1;2* quadruple mutant (Fig. 4). The majority (60.7±7.08% SE, *n*=150) of smaller daughter cells produced after asymmetric division failed to express *TMM* in 9-d-old cotyledons (Fig. 4C–E), consistent with a loss in meristemoid cell fate. In addition, expression from a *proFAMA:GFP* transcriptional fusion was absent from some mature single guard cells in the quadruple mutant (Fig. 4F–H), indicating that some sGCs lose their fate. These findings suggest that abnormal endoreplication is associated with a disruption in cell fate as well as a decrease in epidermal cell number.

### Increased GC size correlates with extensive chloroplast replication

**Fig. 5.**
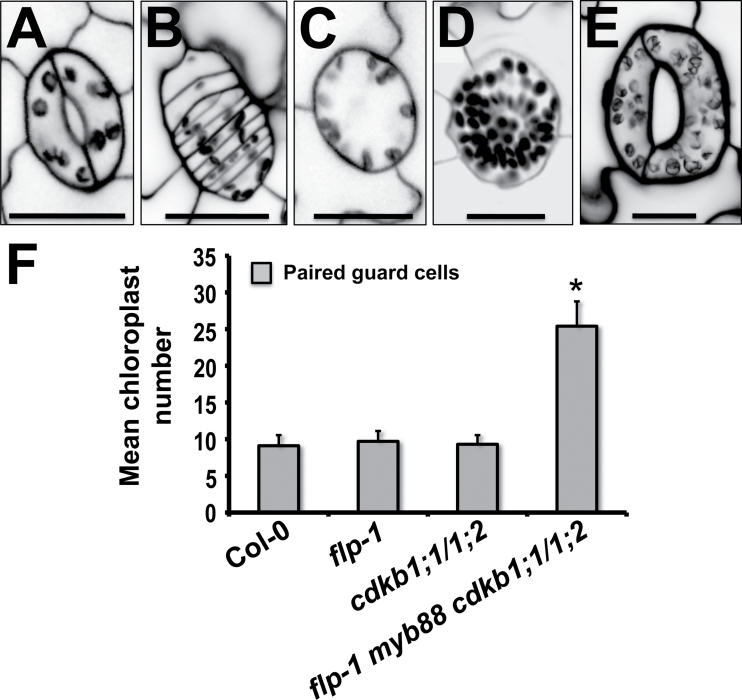
Combined loss of *FLP/MYB88* and *CDKB1;1/1;2* function increases chloroplast number in GCs. (A–E) Comparison of chloroplast number in guard cells as a function of genotype. Propidium iodide staining, confocal microscopy. (A) Wild type. (B) *flp-1 myb88* double mutant. (C) *cdkb1;1 cdkb1;2* double mutant. (D) Single guard cell of the *flp-1 myb88 cdkb1;1 cdkb1;2* quadruple mutant. (E) Abnormally-shaped stoma in the quadruple mutant. (F) Mean chloroplast number in paired guard cells of different genotypes (*n*=10 guard cells per genotype). Bars, means ±SD. Star shows value significantly different from the wild type (*P* <0.001). 20-d-old cotyledons. (A–E) Scale bars=20 µm.

In addition to the larger size and abnormal shape of guard cells in the *flp-1 myb88 cdkb1;1 cdkb1;2* quadruple mutant, this genotype also displayed high numbers of chloroplasts in stomata (Fig. 5). *Arabidopsis* stomata normally contain about 10 chloroplasts per guard cell (Pyke and Leech, 1994). Comparable numbers were present in the *flp-1, flp-1 myb88*, and *cdkb1;1 cdkb1;2* mutant backgrounds (Fig. 5A–C, F). By contrast, mean chloroplast number in stomata with paired guard cells in the *flp-1 myb88 cdkb1;1 cdkb1;2* quadruple mutant was more than double that of the wild type (25.4±3.37 SD) (Fig. 5D–F). Single GCs in the quadruple mutant also contained many chloroplasts (30.2±7.97 SD, *n*=25) (Fig. 5D). Thus abnormal guard cell endoreplication increases plastid number.

### FLP and MYB88 do not affect leaf endoreduplication overall

The *CDKB1;1* dominant negative mutant N161 (*pro35S: CDKB1;1 N161*) promotes endoreduplication in pavement cells as well as in other cells in the cotyledon (Boudolf *et al.*, 2004*b*). Because the loss of *FLP/MYB88* function promotes endoreduplication in the stomatal lineage in a sensitized background, it was probed whether various mutant combinations also affect the degree of endoreduplication at the level of the whole organ. Using nuclei isolated from first leaves harvested 21 d after germination, ploidy distributions were analysed by flow cytometry in control (Col-0) as well as in different mutant backgrounds, i.e. in the *flp-1 myb88* and *cdkb1;1 cdkb1;2* double mutants, and in the *flp-1 myb88 cdkb1;1 cdkb1;2* quadruple mutant. Ploidy levels and their distributions were mostly similar between all four genotypes (see Supplementary Fig. S5 at *JXB* online). However, genotypes lacking CDKB1 function (i.e*. cdkb1* double mutants as well as the quadruple mutant), showed 32C peaks, albeit small, and also had 16C peaks (see Supplementary Fig. S5C, D at *JXB* online).

Although it is likely that endoreplicated nuclei from single guard cells were present in selected flow cytometry samples, presumably this fraction was small compared with the number of nuclei present throughout the leaf (including pavement cells which are highly endoreplicated). Thus FLP and MYB88 do not normally influence endoreduplication in whole leaves, a result consistent with the prominent expression of both MYBs only in the stomatal cell lineage in the cotyledon epidermis, as well as with the lack of any obvious increase in plastid number in other epidermal cells.

### Variable expression of sets of G1-to-S phase genes in the quadruple mutant

Since the quadruple mutant blocks mitosis, the expression of *Arabidopsis* genes known to act in the G1-to-S phase and during endoreplication were analysed in this loss-of-function background. Many G1-to-S phase genes in the quadruple mutant showed no difference in expression levels compared with the wild type. These included *CELL DIVISION CYCLE6* genes (*CDC6*s)*, CDC10 TARGET1* genes (*CDT1*s)*, CDKA;1*, as well as the *CCS52A2/FZR1* and *CCS52A1/FZR2* genes which are required for endoreplication as activators of the Anaphase-Promoting Complex/Cyclosome (APC/C) (see Supplementary Fig. S6 at *JXB* online; Lammens *et al.*, 2008; Larson-Rabin *et al.*, 2009). However, another set of genes was much more highly expressed in the quadruple mutant compared with the wild type. This set included six G1-to-S phase entry genes: (i) *MINI* *-CHROMOSOME MAINTENANCE 2* (*MCM2)*, (ii) *MCM5*, (iii–v) *ORIGIN RECOGNITION COMPLEX 1a* (*ORC1a)*, *ORC2*, *ORC3*, and (vi) *PROLIFERATING CELL NUCLEAR ANTIGEN1* (*PCNA1*) (see Supplementary Fig. S6 at *JXB* online).

Endoreplication initiation is also regulated by atypical E2F repressors, which are characterized by two DNA binding domains, a lack of binding to DIMERIZATION PARTNER (DP) proteins, and the absence of a RETINOBLASTOMA (RB) protein-binding domain (Lammens *et al.*, 2009). Gene expression levels corresponding to a set of E2F related proteins that included typical E2Fs (E2Fa, E2Fb, E2Fc) as well as atypical E2Fs (E2Fd/DEL2, E2Fe/DEL1, E2Ff/DEL3) were therefore quantified in 15-d-old wild-typ*e, flp-1 myb88, cdkb1;1 cdkb1;2* double mutants, and in the quadruple mutant cotyledons. While five of these E2Fs showed no significant differences in expression in the four genotypes, the atypical E2Ff/DEL3 was strongly expressed in the quadruple mutant (see Supplementary Fig. S7 at *JXB* online). Thus E2Ff probably restricts endoreplication, at least in some cell types in the epidermis of wild-type cotyledons.

## Discussion

### FLP conditionally limits guard cell size

The relatively small size of stomata is likely to be adaptive in regulating shoot–atmosphere gas exchange by allowing rapid pore opening and closing. This facilitates rapid stomatal responses to environmental parameters such as local and transient fluctuations in light intensity.

Stomatal size directly correlates with ploidy levels of whole plants. For example, mean stomatal length in natural hexaploid and octoploid *Coffea* plants were more than 50% longer than in diploids (Mishra, 1997). Stomatal size also increases with genome dosage in diploid versus triploid and tetraploid lines in *Arabidopsis* Col and C24 ecotypes (Miller *et al.*, 2012). However, stomata do not normally endoreplicate, consistent with their substantively reduced size compared with other cell types (Melaragno *et al.*, 1993).

*FLP* restricts divisions and enforces patterning in *Arabidopsis* stomatal development by ensuring that only one symmetric division occurs before stomata form (Lai *et al.*, 2005). It is shown here that the paralogous FLP and MYB88 transcription factors can also limit guard cell endoreplication, and thus size but only when the ability of GMCs to divide is blocked.

Whereas oryzalin only arrests GMCs in G2/M in wild-type plants, in *flp* mutants, oryzalin switches GMCs from mitotic cycling to endocycling. These data indicate that GC endoreduplication results from the disruption of microtubule arrays that control both nuclear and cell division.

A similar phenotype arises when CDKB1;1 function is lost. Normally, in addition to promoting the symmetric division of guard mother cells, CDKB1;1 negatively regulates when endocycling starts (Boudolf *et al.*, 2009). While endocycling is pervasive in many tissues and organs in *cdkb1* mutants, it is notably absent from guard cells, even though *CDKB1;1* is expressed late in the stomatal cell lineage (Boudolf *et al.*, 2004*b*). Thus, the lack of endoreplication in *cdkb1;1 cdkb1;2* guard cells, but its presence when FLP function is lost, shows that FLP can conditionally restrict stomatal endocycling and enlargement.

### Direct versus indirect regulation of endoreplication

Mutations in mitotic *CYCLINA2* (*CYCA2*) genes precociously induce and extend the duration of endocycling throughout the shoot (Imai *et al.*, 2006; Vanneste *et al.*, 2011). Endocycling also occurs in *Arabidopsis* cotyledons when the CDKB1;1-CYCA2;3 mitotic complex is inactivated by the APC/C complex (Boudolf *et al.*, 2009). In addition to *CDKB1*, *CYCA2* genes are also expressed late in stomatal development. CYCA2:3 can also promote the last mitosis in stomatal development and its loss of function also induces the formation of single guard cells, but not guard cell endoreplication (Boudolf *et al.*, 2004*a*; Vanneste *et al.*, 2011). Both the *CDKB1;1* and *CYCA2;3* genes are direct transcriptional targets of *FLP/MYB88* (Xie *et al.*, 2010; Vanneste *et al.*, 2011). It remains to be seen whether the loss of *CYCA2* function might also ectopically activate endoreplication, such as in a *flp myb88* double mutant background.

Unlike FLP/MYB88, some transcription factors directly regulate endocycling. For example, the loss of function of *GTL1*, which encodes an *Arabidopsis* trihelix transcription factor, induces extra rounds of endocycling that prolong trichome growth (Breuer *et al.*, 2009). In *Drosophila*, the *ESCARGOT* gene, which encodes a zinc finger transcription factor, induces diploid larval histoblasts to undergo endoreplication at the appropriate developmental stage (Hayashi, 1996). This comparison highlights the relatively indirect role that FLP plays in only conditionally restricting endoreplication.

### FLP and broader regulation of balance between mitosis and endocycling

Although FLP directly targets the DNA replication licensing factor genes, *CDC6a* and *CDC6b*, no changes in their expression levels were detected in *flp myb88*, a result that might reflect the ubiquitous functions of *CDC6* genes. However, the expression of other genes that regulate replication, such as *PCNA1* and some *ORC*s and *MCM*s, was significantly up-regulated in the *flp-1 myb88 cdkb1;1 cdkb1;2* quadruple mutant, but not in either double mutant (*flp-1 myb88* or *cdkb1;1 cdkb1;2*), profiles consistent with guard cell endoreplication only occurring in *flp myb88* after a mitotic block.

Notably, FLP/MYB88 bind specifically to a *cis*-element present in the promoter regions of some potential target genes that overlaps with E2F. For example, FLP/MYB88 directly target and regulate the expression of G1-to-S phase genes which are themselves E2F targets (including *CDC6a*, *CDC6b*, *CYCA2;3*, and *CDKB1;1*) (Xie *et al.*, 2010; Vanneste *et al.*, 2011). Moreover, all G1-to-S phase genes found to be highly expressed in a *flp myb88 cdkb1;1 cdkb1;2* background, such as *MCM2*, *MCM5*, *ORC1a*, *ORC2*, *ORC3,* and *PCNA1*, are known targets of E2F. Thus FLP/MYB88 and E2F probably co-ordinately regulate the expression of cell cycle genes that function in G1-to-S phase, especially those that act later in stomatal development. However, it cannot be ruled out that high expression levels of some G1-to-S phase genes might only be an indirect effect with respect to FLP/MYB88. In addition, FLP/MYB88 might only act conditionally in limiting cell cycling when mitosis itself is restricted.

The *Drosophila* MYB protein, DmMYB, is a component of a multisubunit transcriptional repressive complex termed dREAM/MMB that includes the E2F and RETINOBLASTOMA proteins (Korenjak *et al.*, 2004). The dREAM complex targets E2F promoter binding sites and represses the transcription of genes regulated by E2F (Georlette *et al.*, 2007; van den Heuvel and Dyson, 2008). Thus, FLP/MYB88 and E2F might act in a similar complex in *Arabidopsis* or might regulate common cell cycle target genes. These relationships might also explain why transcription levels of S-phase entry genes increase in a *flp myb88 cdkb1;1 cdkb1;2* background even though many of these genes are direct targets of E2F but not FLP.

The high expression of *E2Ff* (an ‘atypical’ *E2F*) in the quadruple mutant is interesting since the mammalian atypical *E2Fs*, *7* and 8, promote endoreplication, whereas the *Arabidopsis E2Fe* inhibits endocycling (Lammens *et al.*, 2008; Chen *et al.*, 2012; Pandit *et al.*, 2012). This raises the possibility that *E2Ff* might contribute to abnormally high levels of endoreplication when both FLP and CDKB1 functions are lost. To date, E2Ff has only been shown to function in restricting cell expansion in *Arabidopsis* by directly targeting cell wall biosynthesis genes, a role that might be consistent with E2Ff contributing to normal guard cell formation (Ramirez-Parra *et al.*, 2004).

A well-studied endoreplication pathway involves the Anaphase-Promoting Complex/Cyclosome (APC/C). Constitutive expression of *CCS52A1/FZR2*, an APC/C activator, is known to result in abnormally large stomata, as does perturbation of APC/C activity (Larson-Rabin *et al.*, 2009; Iwata *et al.*, 2011; Komaki and Sugimoto, 2012).

APC/C activity is required to initiate endoreduplication. APC/C activator genes, such as *CDH1* (also referred to as *FZR* or *CCS52A*) promote endocycle onset and progression (Larson-Rabin *et al.*, 2009). The initiation of endocycling can also be induced when mitotic cyclins are degraded (Shcherbata *et al.*, 2004; Narbonne-Reveau *et al.*, 2008; Boudolf *et al.*, 2009; Eloy *et al.*, 2012). The activation of APC/C from late G2 to early mitosis requires its interaction with CELL DIVISION CYCLE 20 (CDC20) (also known as FIZZY (FZY)). Later during anaphase to S phase, APC/C remains active by interacting with CELL CYCLE SWITCH 52 (CCS52) (also known as CDC20 HOMOLOG 1 (CDH1)/FIZZY-RELATED (FZR)) (Pesin and Orr-Weaver, 2008).

The APC/C activator *CCS52A1* has been shown to regulate the stability of the CYCA2;3 protein which restricts leaf endoreplication (Boudolf *et al.*, 2009). Since *FLP/MYB88* also directly limit the expression of *CYCA2;3* and *CDKB1;1*, their combined control is also likely to restrict endoreplication in the stomatal pathway (Boudolf *et al.*, 2009; Xie *et al.*, 2010; Vanneste *et al.*, 2011).

### CDKB1 promotes pavement cell formation but restricts their size

The combined loss of CDKB1 and FLP functions leads to smaller leaves and cotyledons as well as an increase in epidermal cell size, especially in non-stomatal epidermal cells, (NSECs), which collectively occupy the greatest share of leaf area. However, the loss of just CDKB1;1 and CDKB1;2 function, whether in a double mutant or in a *CDKB1;1.N161* dominant negative construct, almost halves the number of pavement cells/NSECs (although the N161 construct does not affect the size of cotyledons, as opposed to leaves) (Boudolf *et al.*, 2004*b*). By contrast, the size of leaves and cotyledons appears to be unaffected in *flp myb88*. Our results are consistent with previous findings that the loss of CDKB1 function is largely responsible for increased epidermal cell size. However, mechanisms underlying the relationship between increased pavement cell size and a reduction in the number of stomatal lineage stem cells formed in *cdkb1* mutants remains to be determined.

### Endoreplication, cell shape, and cell fate

Stomatal endoreplication in a *flp myb88 cdkb1;1 cdkb1;2* quadruple mutant background is accompanied by abnormal changes in guard cell shape as well as size. Pavement cells appear to maintain a normal morphology consistent with the confinement of *FLP/MYB88* expression to developing stomata in the leaf epidermis. However, many guard cells in this quadruple mutant expand out of the plane of the epidermis and grow and twist over each other resulting in the loss of stomatal bilateral symmetry.

Abnormal and excessive endoreplication has been shown to be associated with the formation of exceptionally large and irregularly shaped cells that harbour mixed guard cell and pavement cell identity but that bear little resemblance to stomata (Iwata *et al.*, 2011). These cells arise when the function of the *OMISSION OF SECOND DIVISION1 (OSD1)* gene, also known as *GIGAS CELL1,* is also lost (Iwata *et al.*, 2011). *OSD1*, which encodes a novel protein, as well as related genes, probably inhibit APC/C function. Thus while both the *osd1* and the *flp* quadruple mutant phenotypes involve endoreplication, the former induces a much more severe shape defect. In the *flp-1 myb88 cdkb1;1 cdkb1;2* quadruple mutant, cells that resemble mature single GCs later fail to express stomatal fate markers. In addition, many meristemoids, which are the smaller daughter cells of asymmetric division in the lineage and whose expression is normally marked by *TMM*, later lose *TMM* expression. In turn, this reduces the total number of stomata that form in the quadruple mutant (compared with the wild-type).

Cell fate disruption in the quadruple mutant is most prominent in mature cotyledons when endoreplication is more extensive. The close relationship between abnormal endoreplication, altered cell shape, and cell fate disruption, highlights the importance of co-ordinating DNA levels during normal cell morphogenesis and fate determination.

### Guard cell chloroplast number and endoreplication

Whereas diploid *Arabidopsis* Col-0 plants have 2C guard cells that exhibit a relatively small range of sizes and number of chloroplasts (Melaragno *et al.*, 1993; Pyke and Leech, 1994), endoreplicated guard cells in the *flp-1 myb88 cdkb1;1 cdkb1;2* quadruple mutant display more than twice as many chloroplasts compared with the wild-type or to *flp-1 myb88* or *cdkb1;1 cdkb1;2* double mutants. Thus, in addition to restricting GMC symmetric division, FLP and MYB88 also conditionally limit plastid replication.

While a relationship between cell size and plastid number has been questioned based upon comparisons of mesophyll and bundle sheath cells in different taxa (Olszewska *et al.*, 1983), numerous data show that chloroplast number increases relative to nuclear DNA levels (Butterfass, 1973). This relationship has been especially well studied in guard cells in plants that have undergone polyploidization (Butterfass,1973). Our results show that local and cell-type-specific endoreplication can also induce excessive but proportional chloroplast replication. The abundance of chloroplasts in guard cells in the *flp-1 myb88 cdkb1;1 cdkb1;2* quadruple mutant is likely to be a direct consequence of endoreplication since the combined loss of FLP and MYB88 function on its own does not increase plastid number. However, it cannot be ruled out that the enhanced number of chloroplasts in the quadruple mutant might result from unknown factors in addition to or instead of endoreplication.

Overall, this work has shown that two MYB transcription factors that enforce a single symmetric division essential for normal stomatal formation can also conditionally restrict endoreplication and chloroplast and nuclear number, as well normally maintain fate and developmental progression throughout the stomatal cell lineage.

## Supplementary data

Supplementary data can be found at *JXB* online.

Supplementary Fig. S1. Stomatal development in the wild type and selected mutants as well as Histone 2B-YFP expression.

Supplementary Fig. S2. Oryzalin treatment does not appear to affect sGC diameter in *cdkb1;1 cdkb1;2* double mutants.

Supplementary Fig. S3. Expression of *TOO MANY MOUTHS* during stomatal and cotyledon differentiation.

Supplementary Fig. S4. Seedling growth defects in the *flp-1 myb88 cdkb1;1 cdkb1;2* quadruple mutant compared with the wild-type as well as to the *flp-1 myb88* and *cdkb1;1 ckdb1;2* double mutants.

Supplementary Fig. S5. Flow cytometry profiles from PI-stained leaves.

Supplementary Fig. S6. Expression levels of cell cycle-related genes in the *flp-1 myb88 cdkb1;1 cdkb1;2* quadruple mutant as well as other mutants.

Supplementary Fig. S7. Expression levels of *E2F* genes in the *flp-1 myb88 cdkb1;1 cdkb1;2* quadruple mutant as well as in other mutants.

Supplementary Table S1. Primers used.

Supplementary Data
