# Supplementary material for: FOUR LIPS and MYB88 conditionally restrict the G1/S transition during stomatal formation
Source: J Exp Bot. 2013 Oct 11;64(16):5207–19. doi: 10.1093/jxb/ert313 (PMC3830495; doi:10.1093/jxb/ert313)
Supplement: Supplementary Data [file supp_64_16_5207__index.html]

FOUR LIPS and MYB88 conditionally restrict the G1/S transition during stomatal formation — FOUR LIPS and MYB88 conditionally restrict the G1/S transition during stomatal formation — Supplementary Data 

# FOUR LIPS and MYB88 conditionally restrict the G1/S transition during stomatal formation

## Supplementary Data

Data files

**Files in this Data Supplement:**

- Supplementary Data - Supplementary Data
