# Supplementary material for: FOUR LIPS and MYB88 conditionally restrict the G1/S transition during stomatal formation
Source: J Exp Bot. 2013 Oct 11;64(16):5207–19. doi: 10.1093/jxb/ert313 (PMC3830495; doi:10.1093/jxb/ert313)
Supplement: Supplementary Data [file supp_ert313_jexbot104620_file001.pdf]

FOUR LIPS and MYB88 conditionally restrict stomatal endoreplication

EunKyoung Lee, Xuguang Liu, Yana Eglit, and Fred David Sack

SUPPLEMENTARY DATA

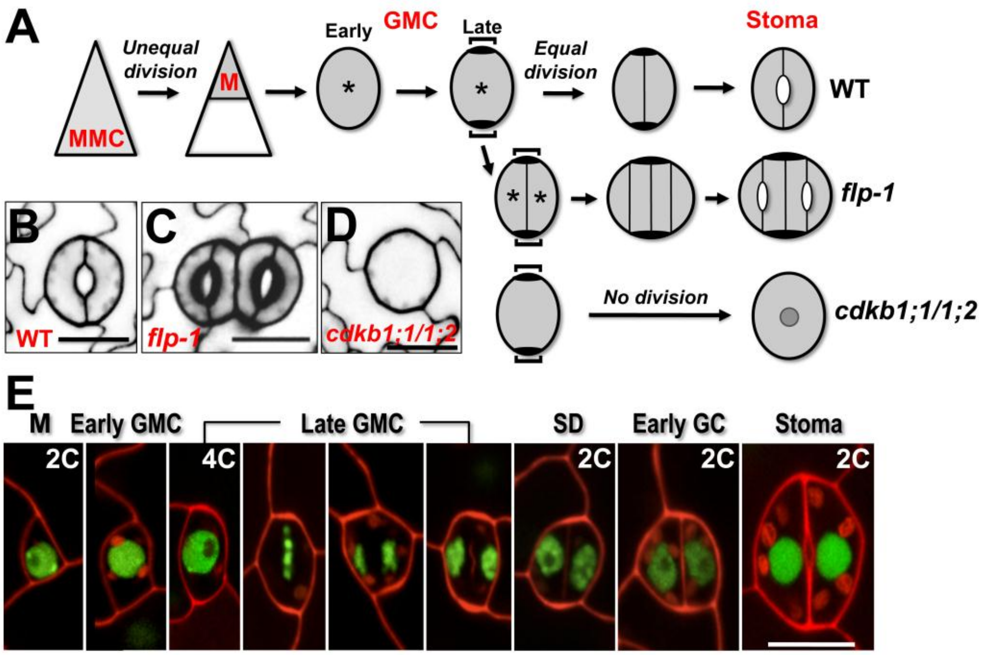

Figure S1. Stomatal development in wild type and selected mutants, as well as Histone 2B-YFP expression. (A) Diagram. Top row: Stomata form via at least one unequal and one equal division. Late GMCs normally develop distinctive end wall thickenings (brackets). MMC, Meristemoid Mother Cell; M, Meristemoid; GMC, Guard Mother Cell (stars). Center row shows development of a *flp-1* stomatal cluster from two daughter cells (stars) that abnormally function as GMCs. Bottom row: Absence of equal division in *cdkb1;1 cdkb1;2* double mutant. (B) Wild-type stoma visualized by fluorescence from propidium iodide. (C) *flp-1* stomatal cluster. (D) *cdkb1;1 cdkb1;2* single guard cell. (E) Histone YFP showing nuclei and divisions during wild-type stomatal development. *pro35S:H2B-YFP* fluorescence shown in green. Bars in (B) to (D) = 20 µm. Bar in (E) = 10 µm with all cells in series shown at same magnification.

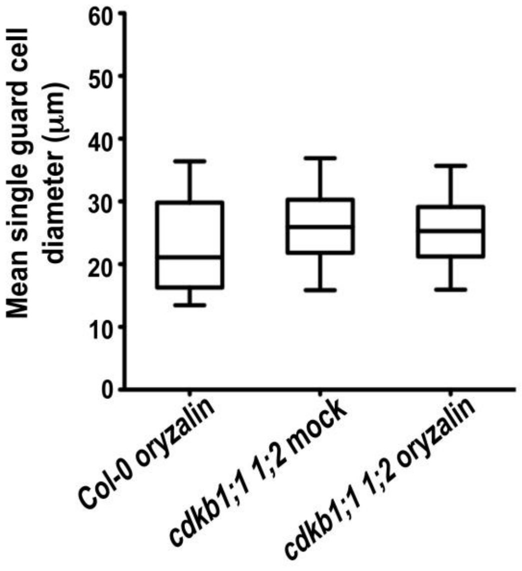

Figure S2. Control showing that, unlike the quadruple mutant, oryzalin treatment does not appear to affect the diameter of single GCs in the *cdkb1;1 cdkb1;2* double mutant. Three day-old *cdkb1;1 cdkb1;2* seedlings were treated with DMSO (mock control) or with 25µM oryzalin for 24 hours, and phenotypes were analyzed 6 days later. The heights of the three bars are proportional to the range of the diameters of single guard cells. Data presented as box plots in which the box encompasses the 25th to 75th percentiles. The horizontal line within each box is the median (50%). Error bars represent the 5th (lower bar) and the 95th (upper bar) percentiles.

Figure S3. Expression of *TOO MANY MOUTHS* during stomatal and cotyledon differentiation. All figures show *proTMM:TMM-GFP* fluorescence that marks stem cells in the *Arabidopsis* stomatal lineage. (A, C, E, G, I) Images of the cotyledon abaxial epidermis of wild-type (WT, left column). (B, D, F, H, J) Images of the cotyledon abaxial epidermis of *flp-1 myb88 cdkb1;1 cdkb1;2* mutant (right column). DAP = days after planting. Upright arrows indicate nearby developing and mature stomata. Stars (*), Single guard cells. Images are at the same magnification. Bar in (f) = 50 µm.

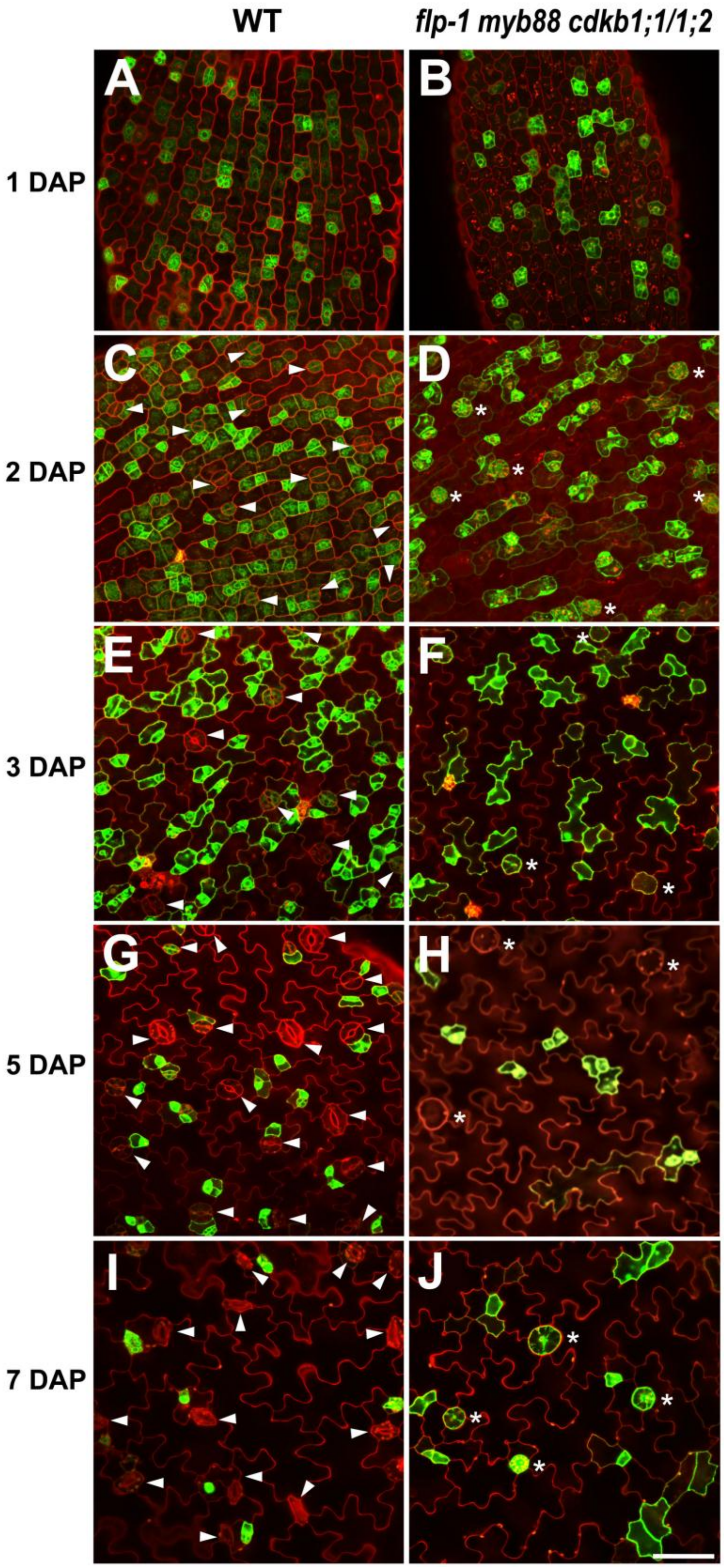

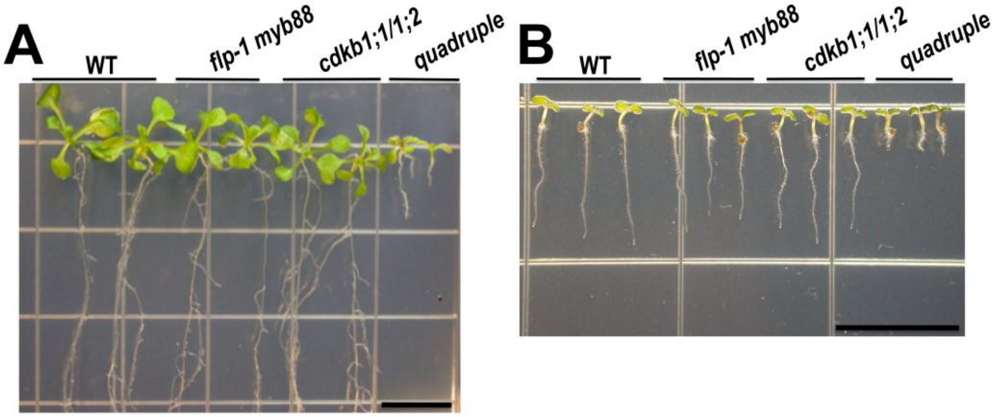

Figure S4. Seedling root and shoot growth defects in the *flp-1 myb88 cdkb1;1 cdkb1;2* quadruple mutant compared to the wild-type as well as to the *flp-1 myb88* and *cdkb1;1 ckdb1;2* double mutants. Quadruple mutant shoots and roots are much reduced in size, and display fewer lateral roots. (A) 20 day-old seedlings. Bar = 10 mm. (B) 4 day-old seedlings. Bar = 10 mm.

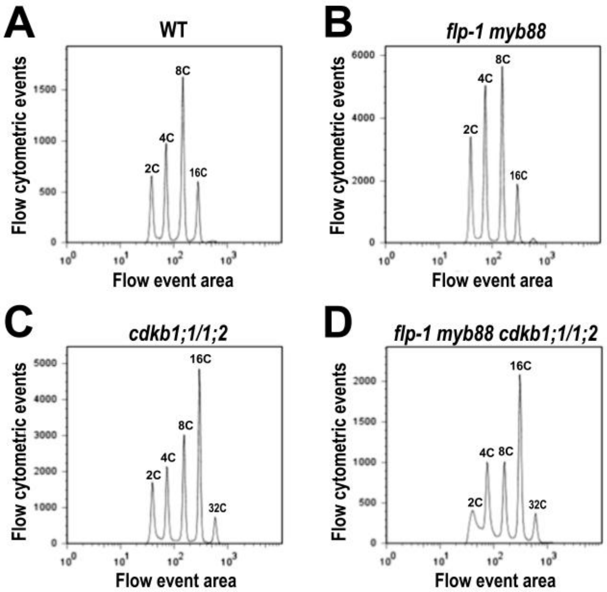

Figure S5. Flow cytometry profiles of cells from propidium iodide-stained leaves.
(A) Wild-type. (B) *flp-1 myb88*. (C) *cdkb1;1 cdkb1;2*. (D) *flp-1 myb88 cdkb1;1 cdkb1;2*.
Ploidy distributions were analyzed by flow cytometry in first leaves harvested 21 days after germination. While all profiles were similar, only genotypes lacking CDKB1 function displayed small 32C peaks.

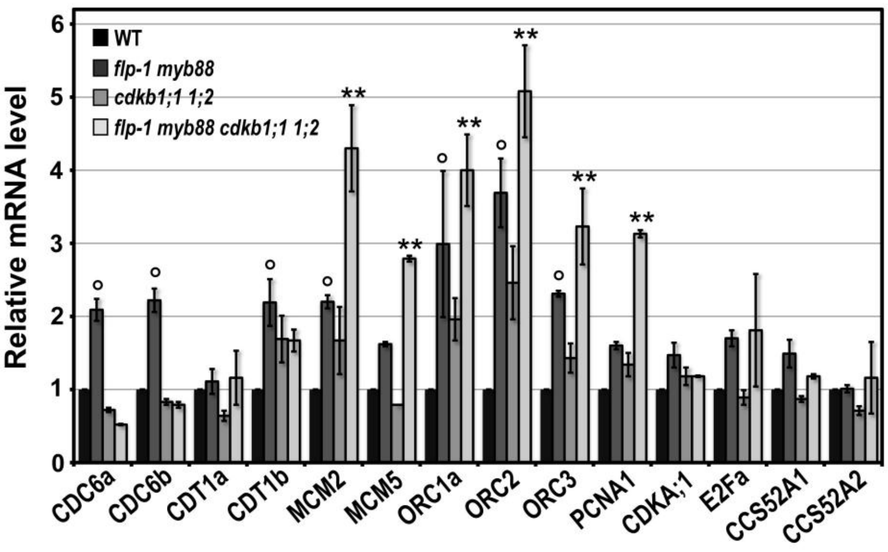

Figure S6. Expression levels of cell cycle-related genes in the *flp-1 myb88 cdkb1;1 cdkb1;2* quadruple mutant as well as other mutants. These data identify several G1-to-S phase genes required for guard cell endoreplication in the quadruple mutant. See Results for full names of genes. RNA levels were measured using quantitative RT-PCR from 15 day-old cotyledons of WT, the *flp-1 myb88* and *cdkb1;1 cdkb1;2* double mutants, and the *flp-1 myb88 cdkb1;1 cdkb1;2* quadruple mutant. Fold levels relative to the wild type were normalized with *ACT2*. Statistically significant differences (P<0.005) between wild-type and *flp-1 myb88* are marked at the top by open circles. The wild-type and the *flp-1 myb88 cdkb1;1 cdkb1;2* quadruple mutant show similar levels of statistical difference (marked by stars). Note the especially high levels of *MCM2*, *MCM5*, *ORC1a, ORC2,* and *ORC3*, as well as *PCNA* in the quadruple mutant background. However, expression levels of other genes, such as *CDT1a* and *CDT1b*, were not different between the wild-type and the quadruple mutant. Such changes might not be detected because they only occur in a small fraction of the cells (*e.g.* related to the stomatal lineage), whereas entire plants were assayed. The involvement of other endoreplication-related genes and other pathways cannot be excluded.

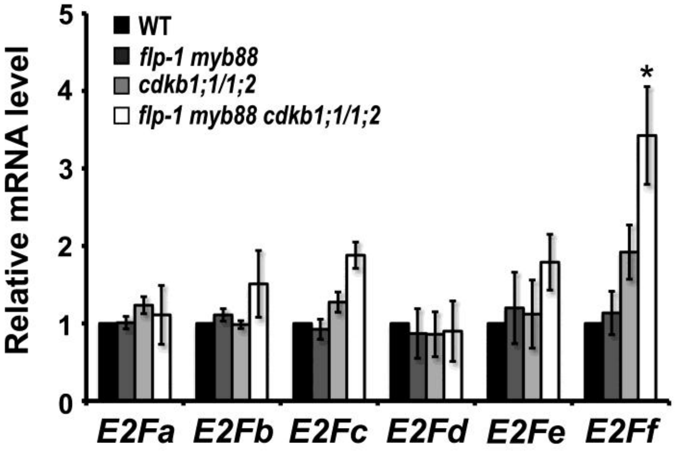

Figure S7. Expression levels of *E2F* genes in the *flp-1 myb88 cdkb1;1 cdkb1;2* quadruple mutant as well as in other mutants. RNA levels were measured using quantitative RT-PCR from 15 day-old cotyledons of WT, the *flp-1 myb88* and *cdkb1;1 cdkb1;2* double mutants, and the *flp-1 myb88 cdkb1;1 cdkb1;2* quadruple mutant. Fold levels relative to the wild type were normalized with *ACT2*. The only statistically significant difference (P<0.005) in expression levels is in *E2Ff* in the quadruple mutant (*).

Table S1. Primers Used.

Gene	Forward primer	Reverse primer
FAMA (promoter)[a]	CACCAAGTATGGTAAGCTAAATTCTGGATC	TGCTATTCGTGGTAGTTGATTATAAACTGC
CDC6a[a]	TGGTGTTGCCTGTGAA	TGACGGTGCCTTAGATAC
CDC6b[a]	AGTCTCTTCCTCAACACC	CTACTCTCAGGCTAACTCTC
CDT1a[a]	GACCTTCATGTTACGCTC	AGCCATCTCTTCCATGAG
CDT1b[a]	AGAACTGCTTCCTGAACC	ATTGGCGTGGACAAGT
CCS52A[b]	ATCAATGCTAATCAATCTCAATCACC	CCAGGAGAATCATCATCAACACC
CCS52A2[b]	TCTTATGCGAGTCTTTTGAAAACGG	GTTCCGCTACTAGTTCCAATAGCC
CDKA;1[b]	ACTGGCCAGAGCATTCGGTATC	TCGGTACCAGAGAGTAACAACCTC
MCM2[a]	CTCGTTGCTCTGTTATTGCTG	ATCCCTGAATGCCATCTTCG-3'
MCM5[b]	AGCTACAGGAGAATCCGGAGGATG	AGATGCCGATCAACTGAGAGAAGC
ORC1A[b]	TCGGTTGTGATCTTGGTGAATGC	GCTGCAGCTTCTGCAATCTGTG
ORC2[a]	GTGGGACAAGAAAATGGTGC	TCTGGGTGTGAAAGTTGGTAC
ORC3[a]	AAGCCAGTTGAGAGTGTTCC	ATGGAAGGCAGTAGAGTTGTG
PCNA1[b]	CGGTGACATTGGAACCGCTAAC	TCACAATTGCATCTTCCGGCTTG
EF1a[b]	TGTAACAAGATGGATGCCACCAC	TCCCTCGAATCCAGAGATTGGC-3'
E2Fb[a]	CCGATGAAAGAGGAAAGCACCG	CGCCTACCTCTGATCGAAACC
E2Fc[a]	GTTAAAGGCTGGGAAACGAATGG	TAAGGTCAAGTGTTCCATCCTCAG
E2Fd[a]	TACAGCCGCAAGGACAAATCTC	TGCAACAAGCCCAATACTCTCC
E2Fe[a]	GTTCAAAACCTGGTTCTCTTCCCC	GCGTCATCAAGGGAGATGATCC
E2Ff[a]	TCCTGAGACTGTATAACCGAGACG	TTGATTCTTCCCTCTTCTTGCCAC
ACT2[a]	CTGGATCGGTGGTTCCATTC	CCTGGACCTGCCTCATCATAC

Sequences are 5' to 3'.

[a] Designed in the present study.

[b] Primers designed according to (Nowack *et al.*, 2012)

Nowack MK, Harashima H, Dissmeyer N, Zhao X, Bouyer D, Weimer AK, De Winter F, Yang F, Schnittger A. 2012. Genetic framework of cyclin-dependent kinase function in Arabidopsis. *Developmental Cell* **22**, 1030–1040.
